# Bis(5-methyl­pyrazine-2-carboxyl­ato-κ^2^ *N*,*O*)nickel(II)

**DOI:** 10.1107/S1600536812024749

**Published:** 2012-06-16

**Authors:** Qi-Ying Shi, Guo-Chun Zhang, Chun-Sheng Zhou, Qi Yang

**Affiliations:** a Department of Chemistry and Chemical Engineering, Shangluo University, Shangluo 726000, Shaanxi, People’s Republic of China; b College of Chemistry and Materials Science, Northwest University, Xi’an 710069, Shaanxi, People’s Republic of China

## Abstract

In the title complex, [Ni(C_6_H_5_O_2_N_2_)_2_], the Ni^II^ atom is situated on an inversion centre and is coordinated in a square-planar geometry by four O atoms and two N atoms of the chelating ligands.

## Related literature

For applications of complexes derived from 2-methyl­pyrazine-5-carb­oxy­lic acid, see: Chapman *et al.* (2002 ▶); Ptasiewicz-Bak & Leciejewicz (2000 ▶); Tanase *et al.* (2006 ▶); Wang *et al.* (2008 ▶) For a related structure, see: Liu *et al.* (2007 ▶). 
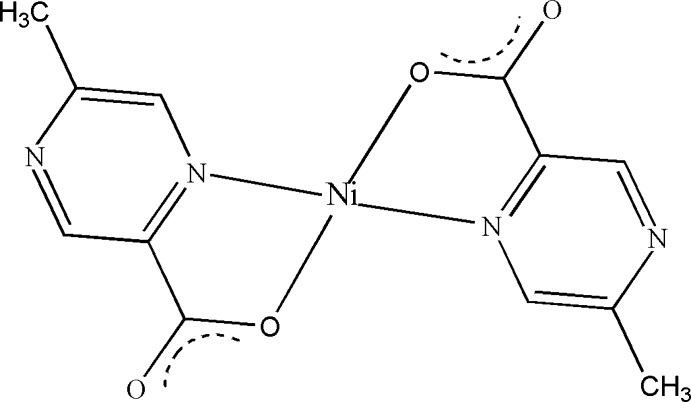


## Experimental

### 

#### Crystal data

[Ni(C_6_H_5_N_2_O_2_)_2_]
*M* *_r_* = 332.95
Monoclinic, 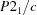

*a* = 11.3098 (19) Å
*b* = 7.6721 (11) Å
*c* = 7.5467 (10) Å
β = 105.647 (2)°
*V* = 630.56 (16) Å^3^
*Z* = 2
Mo *K*α radiation
μ = 1.56 mm^−1^
*T* = 298 K
0.42 × 0.31 × 0.19 mm

#### Data collection

Bruker APEXII CCD diffractometer
Absorption correction: multi-scan (*SADABS*; Sheldrick, 1996 ▶) *T*_min_ = 0.560, *T*_max_ = 0.756
2875 measured reflections
1105 independent reflections
827 reflections with *I* > 2σ(*I*)
*R*_int_ = 0.057

#### Refinement

*R*[*F* ^2^ > 2σ(*F* ^2^)] = 0.062
*wR*(*F* ^2^) = 0.176
*S* = 1.03
1105 reflections
97 parameters
H-atom parameters constrained
Δρ_max_ = 1.34 e Å^−3^
Δρ_min_ = −1.37 e Å^−3^

### 

Data collection: *APEX2* (Bruker, 2004 ▶); cell refinement: *SAINT* (Bruker, 2004 ▶); data reduction: *SAINT*; program(s) used to solve structure: *SHELXS97* (Sheldrick, 2008 ▶); program(s) used to refine structure: *SHELXL97* (Sheldrick, 2008 ▶); molecular graphics: *SHELXTL* (Sheldrick, 2008 ▶); software used to prepare material for publication: *SHELXTL*.

## Supplementary Material

Crystal structure: contains datablock(s) I, global. DOI: 10.1107/S1600536812024749/ru2036sup1.cif

Structure factors: contains datablock(s) I. DOI: 10.1107/S1600536812024749/ru2036Isup2.hkl

Additional supplementary materials:  crystallographic information; 3D view; checkCIF report

## Figures and Tables

**Fig. 1.**
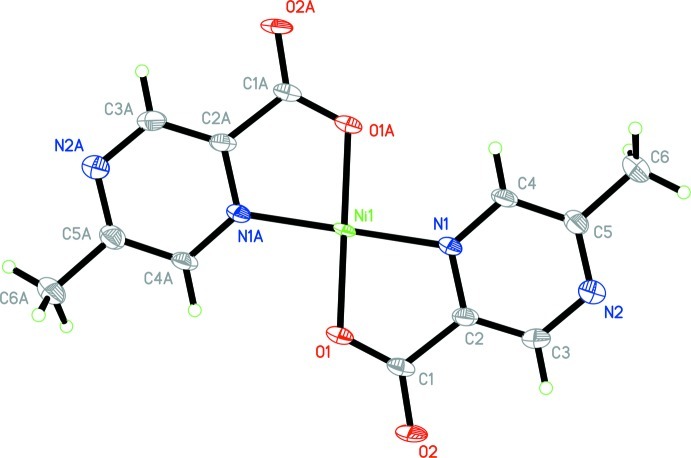
A view of the molecular structure of (I) with the atom-labling scheme. Displacement ellipsoids are drawn at the 30% probability level and H atoms are shown as small spheres of arbitrary radii.

**Fig. 2.**
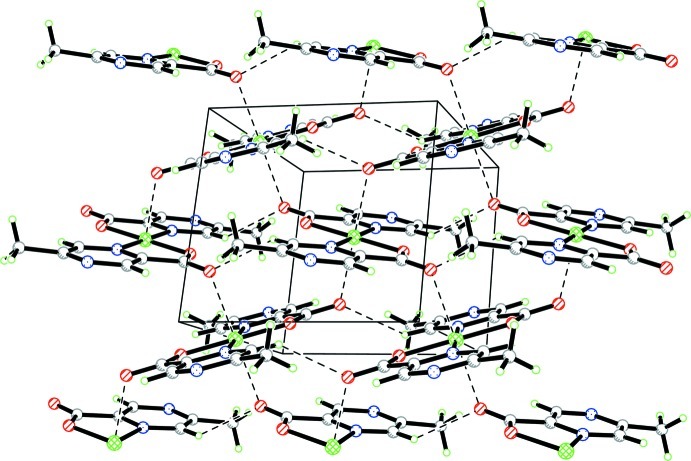
Two dimensional layer sructure of (I)

## supplementary crystallographic information

### Comment

Since the mononuclear complex [Cu(mpca)_2_(H_2_O)3H_2_O](Hmpca = 2-methylpyrazine-5-carboxylic acid) was reported by Leciejewicz, many complexes based on the Hmpca have been prepared. The complex of Hmpca have been extensively investigated and have often been considered for practical use as a class of functional materials. In this paper, we report on the synthesis and characterization of [Ni(mpca)_2_]_n_.

Single-crystal analysis shows the complex crystallizes in monoclinic space group *P*2_1_/*c* and exists as a two-dimensional geometry. As shown in Figure 1, Ni1 is four-coordinated by two oxygen atoms and two nitrogen atoms from two mpca^-^ ligands, displaying a square planar coordination geometry with Ni1—O1 = 1.947 (3) Å and Ni1—N1 = 1.977 (4) Å. The weak coordiantion between Ni1 and O2, which from the adjacent mpca^-^ igand, result in the formation of a distorted octahedral geometry for nickle atom (Ni1—O2=2.509 (2) Å). Then the complex is further extend into a two-dimensional layer structure, see Figure 2.

### Experimental

A mixture of NiCl_2_.6H_2_O (0.238 g, 1 mmol), Hmpca (0.304 g, 1 mmol) and distilled H_2_O (6 ml) was sealed in a 15 ml Teflon-lined stainless steel vessel, which was heated at 120°C for 3 days and then cooled to room temperature at a rate of 5°C/h. Red crystals were obtained, washed with ethanol (yield 43% based on Ni).

### Refinement

The H atoms of C atoms were positioned geometrically and refined with a riding model, with C—H = 0.93 Å and *U*_iso_(H) = 1.2*U*_eq_(C). The water H atoms were located in difference Fourier maps, and were refined with distance restraints of O—H = 0.85±0.02 Å and H^···^H = 1.39±0.02 Å.

### Crystal data

**Table:**

[Ni(C_6_H_5_N_2_O_2_)_2_]	*F*(000) = 340
*M**_r_* = 332.95	*D*_x_ = 1.754 Mg m^−^^3^*D*_m_ = 1.754 Mg m^−^^3^*D*_m_ measured by not measured
Monoclinic, *P*2_1_/*c*	Mo *K*α radiation, λ = 0.71073 Å
Hall symbol: -P 2ybc	Cell parameters from 2220 reflections
*a* = 11.3098 (19) Å	θ = 1.3–24.1°
*b* = 7.6721 (11) Å	µ = 1.56 mm^−^^1^
*c* = 7.5467 (10) Å	*T* = 298 K
β = 105.647 (2)°	Block, green
*V* = 630.56 (16) Å^3^	0.42 × 0.31 × 0.19 mm
*Z* = 2	

### Data collection

**Table:**

Bruker APEXII CCD diffractometer	1105 independent reflections
Radiation source: fine-focus sealed tube	827 reflections with *I* > 2σ(*I*)
Graphite monochromator	*R*_int_ = 0.057
φ and ω scans	θ_max_ = 25.0°, θ_min_ = 1.9°
Absorption correction: multi-scan (*SADABS*; Sheldrick, 1996)	*h* = −13→10
*T*_min_ = 0.560, *T*_max_ = 0.756	*k* = −9→6
2875 measured reflections	*l* = −8→8

### Refinement

**Table:**

Refinement on *F*^2^	Primary atom site location: structure-invariant direct methods
Least-squares matrix: full	Secondary atom site location: difference Fourier map
*R*[*F*^2^ > 2σ(*F*^2^)] = 0.062	Hydrogen site location: inferred from neighbouring sites
*wR*(*F*^2^) = 0.176	H-atom parameters constrained
*S* = 1.03	*w* = 1/[σ^2^(*F*_o_^2^) + (0.121*P*)^2^ + 0.167*P*] where *P* = (*F*_o_^2^ + 2*F*_c_^2^)/3
1105 reflections	(Δ/σ)_max_ < 0.001
97 parameters	Δρ_max_ = 1.34 e Å^−^^3^
0 restraints	Δρ_min_ = −1.37 e Å^−^^3^

### Special details

**Table:**

Geometry. All e.s.d.'s (except the e.s.d. in the dihedral angle between two l.s. planes) are estimated using the full covariance matrix. The cell e.s.d.'s are taken into account individually in the estimation of e.s.d.'s in distances, angles and torsion angles; correlations between e.s.d.'s in cell parameters are only used when they are defined by crystal symmetry. An approximate (isotropic) treatment of cell e.s.d.'s is used for estimating e.s.d.'s involving l.s. planes.
Refinement. Refinement of *F*^2^ against ALL reflections. The weighted *R*-factor *wR* and goodness of fit *S* are based on *F*^2^, conventional *R*-factors *R* are based on *F*, with *F* set to zero for negative *F*^2^. The threshold expression of *F*^2^ > σ(*F*^2^) is used only for calculating *R*-factors(gt) *etc*. and is not relevant to the choice of reflections for refinement. *R*-factors based on *F*^2^ are statistically about twice as large as those based on *F*, and *R*- factors based on ALL data will be even larger.

### Fractional atomic coordinates and isotropic or equivalent isotropic displacement parameters (Å^2^)

**Table:**

	*x*	*y*	*z*	*U*_iso_*/*U*_eq_	
Ni1	0.5000	0.5000	0.5000	0.0317 (4)	
N1	0.3510 (4)	0.4618 (5)	0.5844 (6)	0.0312 (10)	
N2	0.1370 (5)	0.4682 (6)	0.6946 (7)	0.0475 (13)	
O1	0.4846 (3)	0.7385 (4)	0.5791 (5)	0.0412 (9)	
O2	0.3624 (4)	0.9088 (5)	0.6943 (5)	0.0485 (10)	
C1	0.3918 (5)	0.7680 (7)	0.6395 (7)	0.0355 (12)	
C2	0.3114 (5)	0.6106 (6)	0.6398 (6)	0.0342 (12)	
C3	0.2062 (5)	0.6111 (7)	0.6967 (8)	0.0463 (14)	
H3	0.1814	0.7151	0.7389	0.056*	
C4	0.2869 (4)	0.3151 (7)	0.5865 (7)	0.0360 (12)	
H4	0.3148	0.2096	0.5523	0.043*	
C5	0.1780 (5)	0.3214 (7)	0.6401 (7)	0.0404 (13)	
C6	0.1017 (5)	0.1600 (8)	0.6309 (8)	0.0529 (15)	
H6A	0.0434	0.1771	0.7011	0.079*	
H6B	0.1541	0.0633	0.6806	0.079*	
H6C	0.0588	0.1362	0.5051	0.079*	

### Atomic displacement parameters (Å^2^)

**Table:**

	*U*^11^	*U*^22^	*U*^33^	*U*^12^	*U*^13^	*U*^23^
Ni1	0.0421 (6)	0.0079 (5)	0.0502 (7)	−0.0023 (3)	0.0210 (4)	−0.0035 (3)
N1	0.039 (2)	0.016 (2)	0.040 (2)	−0.0016 (17)	0.0133 (18)	0.0004 (16)
N2	0.052 (3)	0.033 (3)	0.062 (3)	−0.003 (2)	0.023 (2)	−0.005 (2)
O1	0.053 (2)	0.0154 (18)	0.059 (2)	−0.0040 (16)	0.0209 (18)	−0.0051 (17)
O2	0.067 (2)	0.014 (2)	0.068 (3)	0.0046 (18)	0.0242 (19)	−0.0060 (17)
C1	0.048 (3)	0.016 (3)	0.041 (3)	0.000 (2)	0.010 (2)	−0.001 (2)
C2	0.046 (3)	0.019 (3)	0.039 (3)	0.001 (2)	0.013 (2)	−0.004 (2)
C3	0.059 (4)	0.026 (3)	0.061 (3)	0.004 (2)	0.027 (3)	−0.007 (2)
C4	0.044 (3)	0.016 (3)	0.048 (3)	−0.001 (2)	0.012 (2)	−0.002 (2)
C5	0.050 (3)	0.030 (3)	0.044 (3)	−0.007 (2)	0.018 (2)	0.001 (2)
C6	0.058 (3)	0.037 (3)	0.067 (4)	−0.015 (3)	0.022 (3)	−0.003 (3)

### Geometric parameters (Å, º)

**Table:**

Ni1—O1	1.947 (3)	C1—C2	1.512 (7)
Ni1—O1^i^	1.947 (3)	C2—C3	1.370 (7)
Ni1—N1	1.977 (4)	C3—H3	0.9300
Ni1—N1^i^	1.977 (4)	C4—C5	1.397 (7)
N1—C2	1.335 (6)	C4—H4	0.9300
N1—C4	1.341 (6)	C5—C6	1.501 (7)
N2—C5	1.325 (7)	C6—H6A	0.9600
N2—C3	1.345 (7)	C6—H6B	0.9600
O1—C1	1.272 (6)	C6—H6C	0.9600
O2—C1	1.233 (6)		
			
O1—Ni1—O1^i^	180.000 (1)	C3—C2—C1	124.9 (5)
O1—Ni1—N1	83.45 (16)	N2—C3—C2	123.1 (5)
O1^i^—Ni1—N1	96.55 (16)	N2—C3—H3	118.5
O1—Ni1—N1^i^	96.55 (16)	C2—C3—H3	118.5
O1^i^—Ni1—N1^i^	83.45 (16)	N1—C4—C5	119.7 (5)
N1—Ni1—N1^i^	180.0	N1—C4—H4	120.1
C2—N1—C4	119.1 (4)	C5—C4—H4	120.1
C2—N1—Ni1	111.2 (3)	N2—C5—C4	122.0 (5)
C4—N1—Ni1	129.7 (4)	N2—C5—C6	118.1 (5)
C5—N2—C3	116.4 (5)	C4—C5—C6	119.9 (5)
C1—O1—Ni1	115.3 (3)	C5—C6—H6A	109.5
O2—C1—O1	126.8 (5)	C5—C6—H6B	109.5
O2—C1—C2	118.9 (4)	H6A—C6—H6B	109.5
O1—C1—C2	114.4 (4)	C5—C6—H6C	109.5
N1—C2—C3	119.6 (5)	H6A—C6—H6C	109.5
N1—C2—C1	115.5 (4)	H6B—C6—H6C	109.5
			
O1—Ni1—N1—C2	3.6 (3)	Ni1—N1—C2—C1	−4.3 (5)
O1^i^—Ni1—N1—C2	−176.4 (3)	O2—C1—C2—N1	−177.9 (4)
N1^i^—Ni1—N1—C2	−75 (100)	O1—C1—C2—N1	2.7 (6)
O1—Ni1—N1—C4	−178.8 (5)	O2—C1—C2—C3	0.1 (8)
O1^i^—Ni1—N1—C4	1.2 (5)	O1—C1—C2—C3	−179.3 (5)
N1^i^—Ni1—N1—C4	102 (100)	C5—N2—C3—C2	2.5 (9)
O1^i^—Ni1—O1—C1	−153 (100)	N1—C2—C3—N2	−2.2 (9)
N1—Ni1—O1—C1	−2.3 (3)	C1—C2—C3—N2	179.9 (5)
N1^i^—Ni1—O1—C1	177.7 (3)	C2—N1—C4—C5	2.2 (7)
Ni1—O1—C1—O2	−178.9 (4)	Ni1—N1—C4—C5	−175.2 (3)
Ni1—O1—C1—C2	0.5 (5)	C3—N2—C5—C4	−0.4 (8)
C4—N1—C2—C3	−0.3 (7)	C3—N2—C5—C6	−178.4 (5)
Ni1—N1—C2—C3	177.6 (4)	N1—C4—C5—N2	−1.9 (8)
C4—N1—C2—C1	177.8 (4)	N1—C4—C5—C6	176.0 (5)

Symmetry code: (i) −*x*+1, −*y*+1, −*z*+1.

## References

[bb1] Bruker (2004). *APEX2* and *SAINT* Bruker AXS Inc., Madison, Wisconsin, USA.

[bb2] Chapman, C. T., Ciurtin, D. M., Smith, M. D. & Loye zur, H. C. (2002). *Solid State Sci.* **4**, 1187–1189.

[bb3] Liu, F.-Y., Shang, R.-L., Du, L., Zhao, Q.-H. & Fang, R.-B. (2007). *Acta Cryst.* E**63**, m120–m122.

[bb4] Ptasiewicz-Bak, H. & Leciejewicz, J. (2000). *Pol. J. Chem.* **74**, 877–883.

[bb5] Sheldrick, G. M. (1996). *SADABS* University of Göttingen, Germany.

[bb6] Sheldrick, G. M. (2008). *Acta Cryst.* A**64**, 112–122. 10.1107/S0108767307043930 18156677

[bb7] Tanase, S., Martin, V. S., Van Albada, G. A., DeGelder, R., Bouwman, E. & Reedijk, J. (2006). *Polyhedron*, **25**, 2967–2975.

[bb8] Wang, F. Q., Mu, W. H., Zheng, X. J., Li, L. C., Fang, D. C. & Jin, L. P. (2008). *Inorg. Chem.* **47**, 5225–5233. 10.1021/ic8001916 18489090

